# mCRPC patients with PSA fluctuations under radioligand therapy have comparable survival benefits relative to patients with sustained PSA decrease

**DOI:** 10.1007/s00259-022-05910-w

**Published:** 2022-07-19

**Authors:** Philipp E. Hartrampf, Ralph A. Bundschuh, Franz-Xaver Weinzierl, Sebastian E. Serfling, Aleksander Kosmala, Anna Katharina Seitz, Hubert Kübler, Andreas K. Buck, Markus Essler, Rudolf A. Werner

**Affiliations:** 1 Department of Nuclear Medicine, University Hospital Wuerzburg, Oberdürrbacherstraße Str. 6, 97080 Würzburg, Germany; 2 Department of Nuclear Medicine, University Hospital Bonn, Venusberg-Campus 1, 53127 Bonn, Germany; 3 Department of Urology and Paediatric Urology, University Hospital Wuerzburg, Oberdürrbacherstraße 6, 97080 Würzburg, Germany; 4 The Russell H Morgan Department of Radiology and Radiological Science, Division of Nuclear Medicine and Molecular Imaging, Johns Hopkins School of Medicine, Baltimore, MD USA

**Keywords:** Radioligand therapy, Late response, Flare phenomenon, PSA, Prostate cancer, PSMA

## Abstract

**Introduction:**

In men with metastatic castration-resistant prostate cancer (mCRPC) scheduled for prostate-specific membrane antigen (PSMA)-targeted radioligand therapy (RLT), biochemical response is assessed based on repeated measurements of prostate-specific antigen (PSA) levels. We aimed to determine overall survival (OS) in patients experiencing sustained PSA increase, decrease, or fluctuations during therapy.

**Materials and methods:**

In this bicentric study, we included 176 mCRPC patients treated with PSMA-directed RLT. PSA levels were determined using blood samples prior to the first RLT and on the admission days for the following cycles. We calculated relative changes in PSA levels compared to baseline. Kaplan–Meier curves as well as log-rank test were used to compare OS of different subgroups, including patients with sustained PSA increase, decrease, or fluctuations (defined as change after initial decrease or increase after the first cycle).

**Results:**

Sixty-one out of one hundred seventy-six (34.7%) patients showed a sustained increase and 86/176 (48.8%) a sustained decrease in PSA levels. PSA fluctuations were observed in the remaining 29/176 (16.5%). In this subgroup, 22/29 experienced initial PSA decrease followed by an increase (7/29, initial increase followed by a decrease). Median OS of patients with sustained decrease in PSA levels was significantly longer when compared to patients with sustained increase of PSA levels (19 vs. 8 months; HR 0.35, 95% CI 0.22–0.56; *P* < 0.001). Patients with PSA fluctuations showed a significantly longer median OS compared to patients with sustained increase of PSA levels (18 vs. 8 months; HR 0.49, 95% CI 0.30–0.80; *P* < 0.01), but no significant difference relative to men with sustained PSA decrease (18 vs. 19 months; HR 1.4, 95% CI 0.78–2.49; *P* = 0.20). In addition, in men experiencing PSA fluctuations, median OS did not differ significantly between patients with initial decrease or initial increase of tumor marker levels (16 vs. 18 months; HR 1.2, 95% CI 0.38–4.05; *P* = 0.68).

**Conclusion:**

Initial increase or decrease of PSA levels is sustained in the majority of patients undergoing RLT. Sustained PSA decrease was linked to prolonged survival and men with PSA fluctuations under treatment experienced comparable survival benefits. As such, transient tumor marker oscillations under RLT should rather not lead to treatment discontinuation, especially in the absence of radiological progression.

**Supplementary Information:**

The online version contains supplementary material available at 10.1007/s00259-022-05910-w.

## Introduction

For patients with metastatic castration-resistant prostate cancer (mCRPC), prostate-specific membrane antigen (PSMA)-targeted radioligand therapy (RLT) has shown survival benefits in recent prospective clinical trials [1, 2], leading to approval by the Food and Drug Administration [3]. Despite those beneficial results, a substantial portion of patients does not respond to therapy, and biomarkers to identify patients prone to treatment failure include regular assessment of prostate-specific antigen (PSA) levels. In this regard, early biochemical response 8 weeks after the first cycle has been advocated to be linked to prolonged overall survival (OS) [4–6]. However, failure to achieve substantial decline of PSA levels does not necessarily imply treatment failure. In this regard, a PSA flare phenomenon has been described for various prostate-specific therapies, e.g., docetaxel, abiraterone, or other therapeutic radionuclides, such as radium-223 dichloride [7–9]. Defined as an initial increase in PSA levels in the first weeks after therapy, followed by a delayed decrease in PSA levels, the outcome in patients with such a PSA flare was similar to those in men exhibiting early biochemical response [7–9]. Flare phenomenon has also been discussed in RLT [10], but appears to be very rare [5]. Nevertheless, previous studies have reported on up to 30% of initial non-PSA responders having a delayed decrease in PSA levels [10, 11].

Enrolling the largest cohort to date for this purpose, we aimed to assess OS in patients experiencing initial and sustained PSA increase, continuous decrease, or fluctuations during therapy (defined as change in PSA levels after initial decrease or increase). We then compared survival among subgroups, thereby allowing to determine whether survival benefits of patients with PSA fluctuations under RLT are comparable to those under sustained PSA decline.

## Material and methods

### Patient cohort

We included 176 patients with mCRPC treated with PSMA-directed RLT at the University Hospital of Würzburg ([^177^Lu]Lu-PSMA I&T, *n* = 89) and Bonn ([^177^Lu]Lu-PSMA-617, *n* = 87). Only men with at least two cycles and continuous RLT were included (Table 1). We have already partially reported on those subjects in [12–14], without a comparison of different subgroups showing sustained biochemical response, failure, or PSA fluctuations. The local ethical committee waived the need for approval due to the retrospective nature of this analysis (20210422 04).

**Table 1:** Patient characteristics. ^#^Comparison between patients treated with [^177^Lu]Lu-PSMA I&T and [^177^Lu]Lu-PSMA-617

	Entire cohort (*n* = 176)	[^177^Lu]Lu-PSMA I&T (*n* = 89)	[^177^Lu]Lu-PSMA-617 (*n* = 87)	*P* value^#^
*Clinical variables*				
Median age at first cycle of PSMA-RLT (years)	71.0 (43.0–90.0)	72.0 (46.0–90.0)	71.0 (43.0–86.0)	0.49
Median treatment cycles per patient	3 (2–10)	3 (2–9)	3 (2–10)	0.56
Median cumulative activity (GBq)	18.9 (7.9–60.1)	18.4 (10.4–54.8)	19.0 (7.9–60.1)	0.53
Median Gleason score	8 (6–10)	9 (6–10)	8 (6–10)	0.75
*Baseline laboratory values*				
Median PSA (ng/ml)	179.0 (0.4–3130)	158.0 (5.0–3130)	191.0 (0.4–2600)	0.49
*Prior treatments (%)*				
Radical prostatectomy	74/176 (42.0)	39/89 (43.8)	35/87 (40.2)	
Primary radiation therapy to the prostate	29/176 (16.5)	14/89 (15.7)	15/87 (17.2)	
Adjuvant radiation therapy	28/176 (15.9)	17/89 (19.1)	11/87 (12.6)	
Salvage radiation therapy	21/176 (11.9)	14/89 (15.7)	7/87 (8.0)	
Anti-hormonal treatment	176/176 (100)	89/89 (100)	87/87 (100)	
Enzalutamide	124/176 (70.5)	62/89 (69.7)	62/87 (71.3)	
Abiraterone	123/176 (69.9)	65/89 (73.0)	58/87 (66.7)	
Chemotherapy	125/176 (71.0)	57/89 (64.0)	68/87 (78.2)	

*PSA*, prostate-specific antigen

### Treatment protocol

For all patients, we applied standard institutional protocols to perform RLT after in-house labeling of PSMA ligands with [^177^Lu], as described for both departments in [12, 15]. In brief, we administered approximately 6.0 GBq of PSMA ligands in 8-week intervals.

### Determination of PSA levels

PSA levels were determined using blood samples prior to the first RLT (cycle 1, day 0) and on the admission days for the following cycles. For the present analysis, we included PSA values up to cycle 4, day 0. Following the recommendations of the Prostate Cancer Clinical Trials Working Group 3 (PCWG3), patients were allocated to different categories [16]. In this regard, response was defined as PSA decrease ≥ 50%, progressive disease (PD) as PSA increase that is ≥ 25% and ≥ 2 ng/mL above the nadir as well as stable disease (SD) which is neither response nor PD. Categories were then as follows: (1) sustained response; (2) response followed by SD; (3) SD; (4) response followed by PD; (5) SD followed by PD; and (6) PD.

Independent of PCWG3 criteria, we also calculated relative changes in PSA levels compared to baseline levels, and categorized them as (a) initial and sustained decrease in PSA levels, or (b) initial and sustained increase in PSA levels (this included patients who received only two cycles of PSMA-RLT, but had unequivocal disease progression or death), or (c) PSA fluctuations during follow-up, characterized by change after initial decrease or increase (after the first cycle). For the subgroup (c), late responders were characterized by an initial increase followed by a decrease in PSA levels.

We then compared survival between all subgroups. OS was defined as the time between first cycle of RLT and date of death.

### Statistical analysis

We used GraphPad Prism version 9.3.0 (GraphPad Software, San Diego, CA, USA). Our descriptive data are presented as median and range in parentheses. Comparisons of patient characteristics of subjects either treated with [^177^Lu]Lu-PSMA-617 or -I&T were performed using Mann–Whitney *U* test. Kaplan–Meier curves and log-rank test were used to compare OS of different subgroups. We present the median survival in months with hazard ratio of death (HR) along with 95% confidence interval (95% CI). We considered a *P* value of less than 0.05 statistically significant.

## Results

### Patient characteristics

The median age of patients at the first cycle of therapy was 71 (43–90) years. The median Gleason score was 8, while the median baseline PSA level was 179.0 (0.4–3130) ng/mL. Overall, patients received a median of three cycles of PSMA-RLT with a median of 18.9 (7.9–60.1) GBq of cumulative administered activity. Forty-two percent of patients received radical prostatectomy as first-line treatment and 16.5% received radiation therapy to the prostate. All patients received anti-hormonal treatment, up to 70.7% had received prior second-line anti-hormonal treatment (enzalutamide, abiraterone), and 71% had received prior chemotherapy. No significant differences were observed between patients treated with [^177^Lu]Lu-PSMA I&T and [^177^Lu]Lu-PSMA-617 (Table 1).

### Patients with PD had shorter overall survival compared to all other patients

**Fig. 1:**
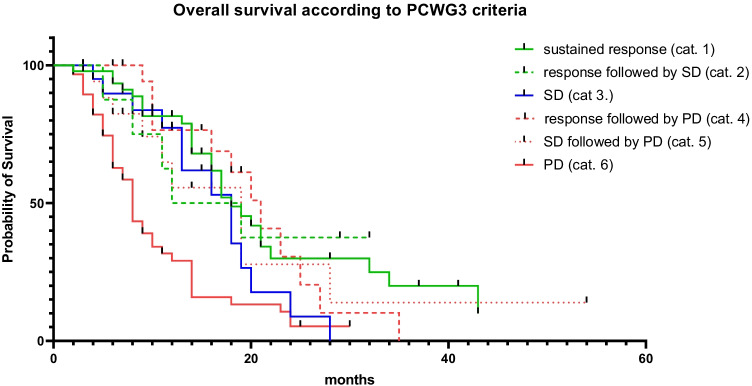
Following the recommendations of the Prostate Cancer Clinical Trials Working Group 3 (PCWG3), patients were allocated to different categories [16], which also took PSA values during follow-up into account. The derived categories were as follows: (1) sustained response, (2) response followed by SD, (3) SD, (4) response followed by PD, (5) SD followed by PD, (6) PD. Median overall survival was significantly shorter for patients in category 6 (PD; 8 months) compared to all other categories (category 1: 18 months, HR 0.36, 95% CI 0.22–0.59, *P* < 0.001; category 2: 15.5 months, HR 0.41, 95% CI 0.21–0.81, *P* = 0.03; category 3: 18 months, HR 0.52, 95% CI 0.30–0.89, *P* = 0.02; category 4: 21 months, HR 0.37, 95% CI 0.22–0.63, *P* < 0.001; category 5: 19 months, HR 0.45, 95% CI 0.25–0.80, *P* = 0.02). There were no significant differences within the categories 1–5 (*P* ≥ 0.11). cat., category; SD, stable disease; PD, progressive disease

According to our six predefined categories following PCWG3 criteria [16], 47/176 patients were allocated to category 1, 8/176 to category 2, 20/176 to category 3, 20/176 to category 4, 18/176 to category 5, and 61/176 to category 6. Two patients had to be excluded due to classification, which did not fall into one of the predefined categories. OS was significantly shorter for patients in category 6 (PD; 8 months) compared to all other categories (category 1: 18 months, HR 0.36, 95% CI 0.22–0.59, *P* < 0.001; category 2: 15.5 months, HR 0.41, 95% CI 0.21–0.81, *P* = 0.03; category 3: 18 months, HR 0.52, 95% CI 0.30–0.89, *P* = 0.02; category 4: 21 months, HR 0.37, 95% CI 0.22–0.63, *P* < 0.001; category 5, 19 months, HR 0.45, 95% CI 0.25–0.80; *P* = 0.02). No significant differences in OS were found when comparing patients within the categories 1–5 (*P* ≥ 0.11) (Fig. 1).

### PSA fluctuations occur frequently, but rate of late responders is low

For the entire cohort, 61/176 (34.7%) patients showed a sustained increase in PSA levels and 86/176 (48.8%) a sustained decrease in PSA levels. The remaining 29 out of 176 men (16.5%) showed PSA fluctuations. Twenty-two out of twenty-nine of those patients showed an initial decrease followed by an increase during follow-up, while the remaining 7 patients were classified as late responders. As such, among the entire cohort, only 4% (7/176) demonstrated late response.

**Fig. 2:**
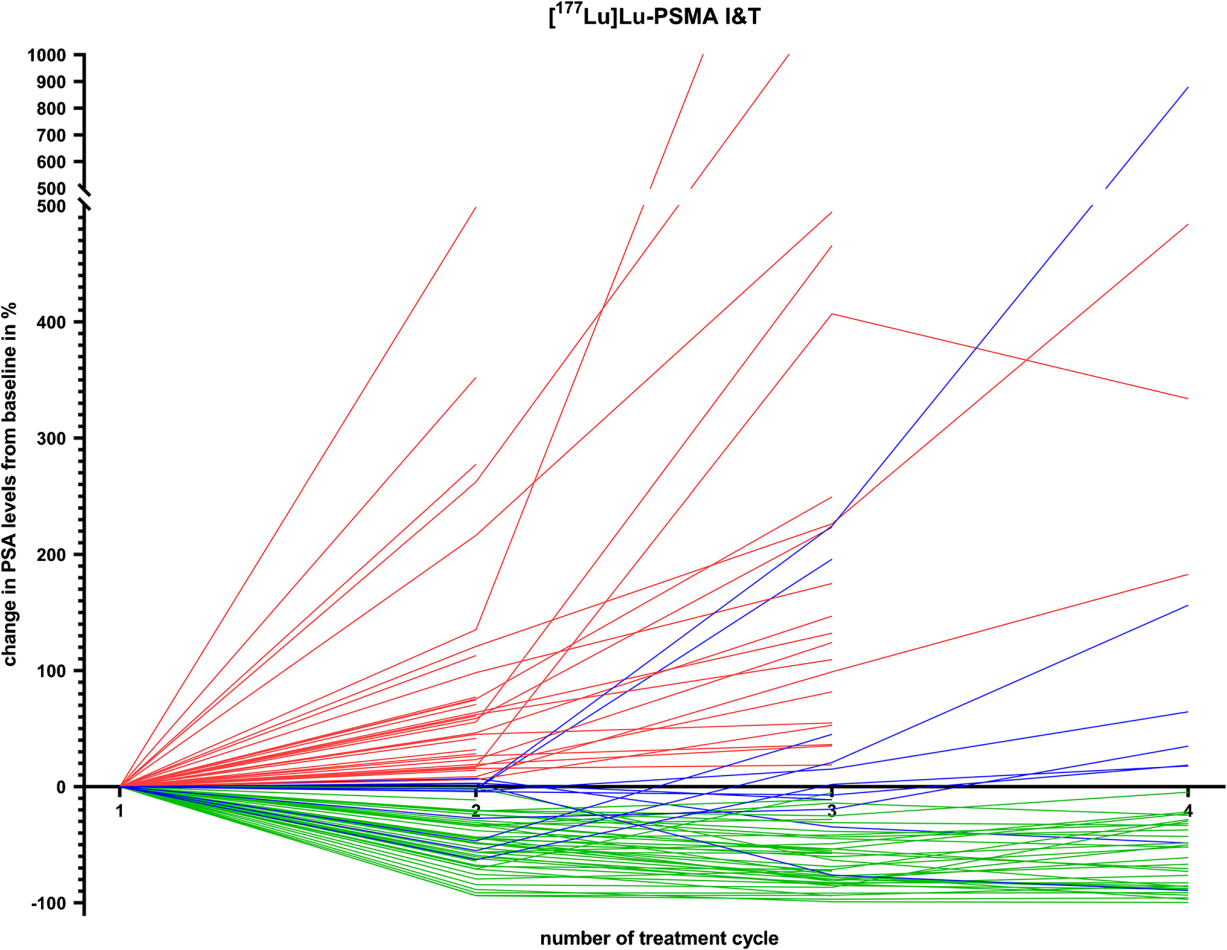
PSA levels over time in patients undergoing radioligand therapy with [^177^Lu]Lu-PSMA I&T. Patients with sustained decrease of PSA levels are marked in green. Patients with sustained increase of PSA levels are marked in red. Patients with PSA fluctuations (blue) were defined as changes over time after initial increase or decrease and thus, are crossing zero line

Similar results were achieved when analyzing patients that received either [^177^Lu]Lu-PSMA-617 or -I&T, with patient’s characteristics demonstrating no significant differences between both groups (*P* ≥ 0.49; Table 1). For individuals treated with the latter radiopharmaceutical, 34/89 (38.2%) patients showed a sustained increase in PSA levels and 44/89 (49.4%) a sustained decrease in PSA levels. The remaining 11/89 (12.4%) showed PSA fluctuations. Of those patients, 8/11 showed an initial decrease followed by increase during follow-up, while 3/11 were late responders. Detailed PSA courses over all treatment cycles for patients treated with [^177^Lu]Lu-PSMA I&T are shown in Fig. 2 (with patients experiencing PSA fluctuations crossing the zero line indicated in blue).

**Fig. 3:**
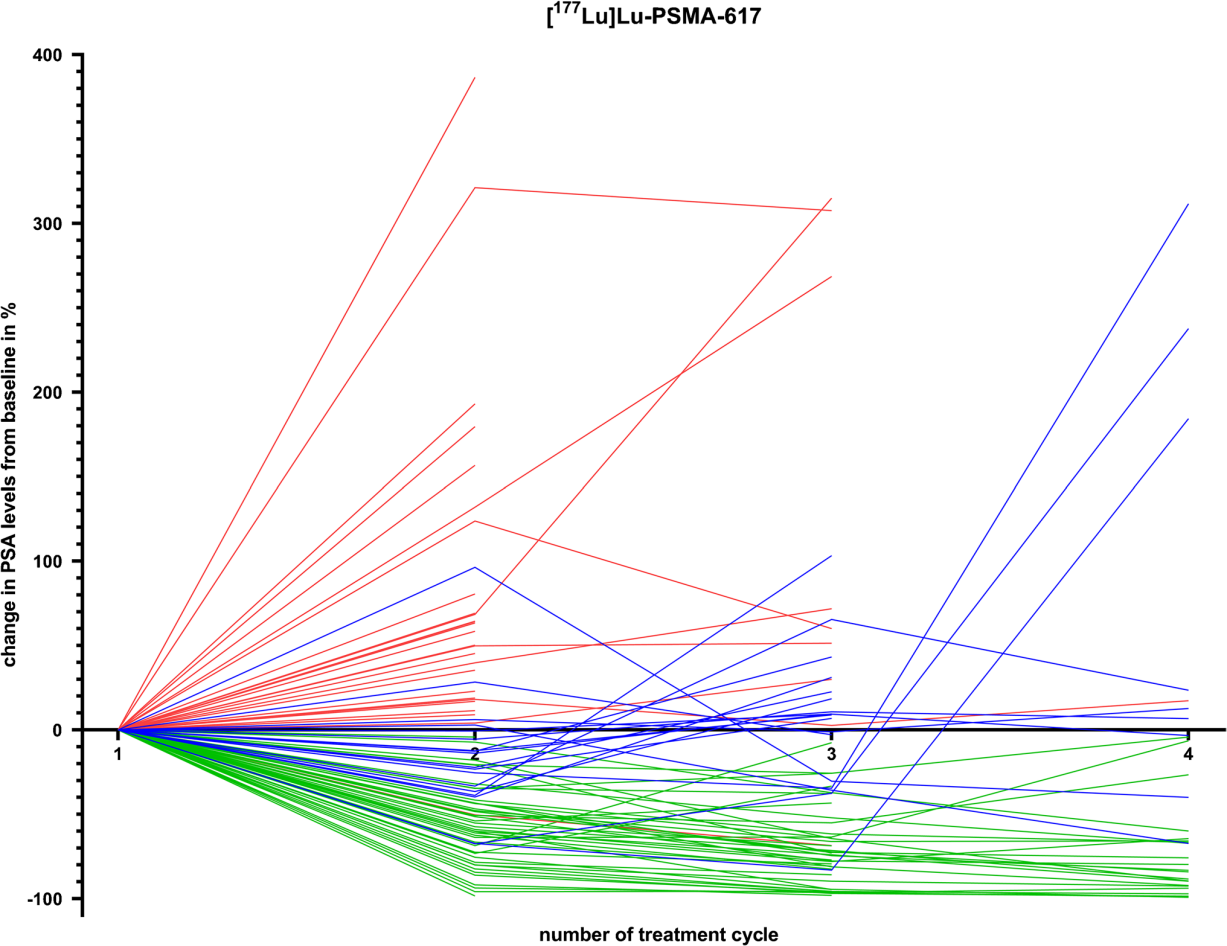
PSA levels over time in patients undergoing radioligand therapy with [^177^Lu]Lu-PSMA-617. Patients with sustained decrease of PSA levels are marked in green. Patients with sustained increase of PSA levels are marked in red. Patients with PSA fluctuations (blue) were defined as changes over time after initial increase or decrease and thus, are crossing zero line

For [^177^Lu]Lu-PSMA-617, 27/87 (31.0%) patients showed a sustained increase and 42/87 (48.3%) a sustained decrease in PSA levels. Eighteen out of eighty-seven (20.7%) showed PSA fluctuations. Of those men, 14/18 showed an initial decrease followed by increase in the later cycles, while the remaining four were late responders. For [^177^Lu]Lu-PSMA-617, a detailed PSA course over all treatment cycles is provided in Fig. 3 (with patients experiencing PSA fluctuations crossing the zero line indicated in blue).

### Survival in men with PSA fluctuations is comparable to those with maintained PSA decrease

**Fig. 4:**
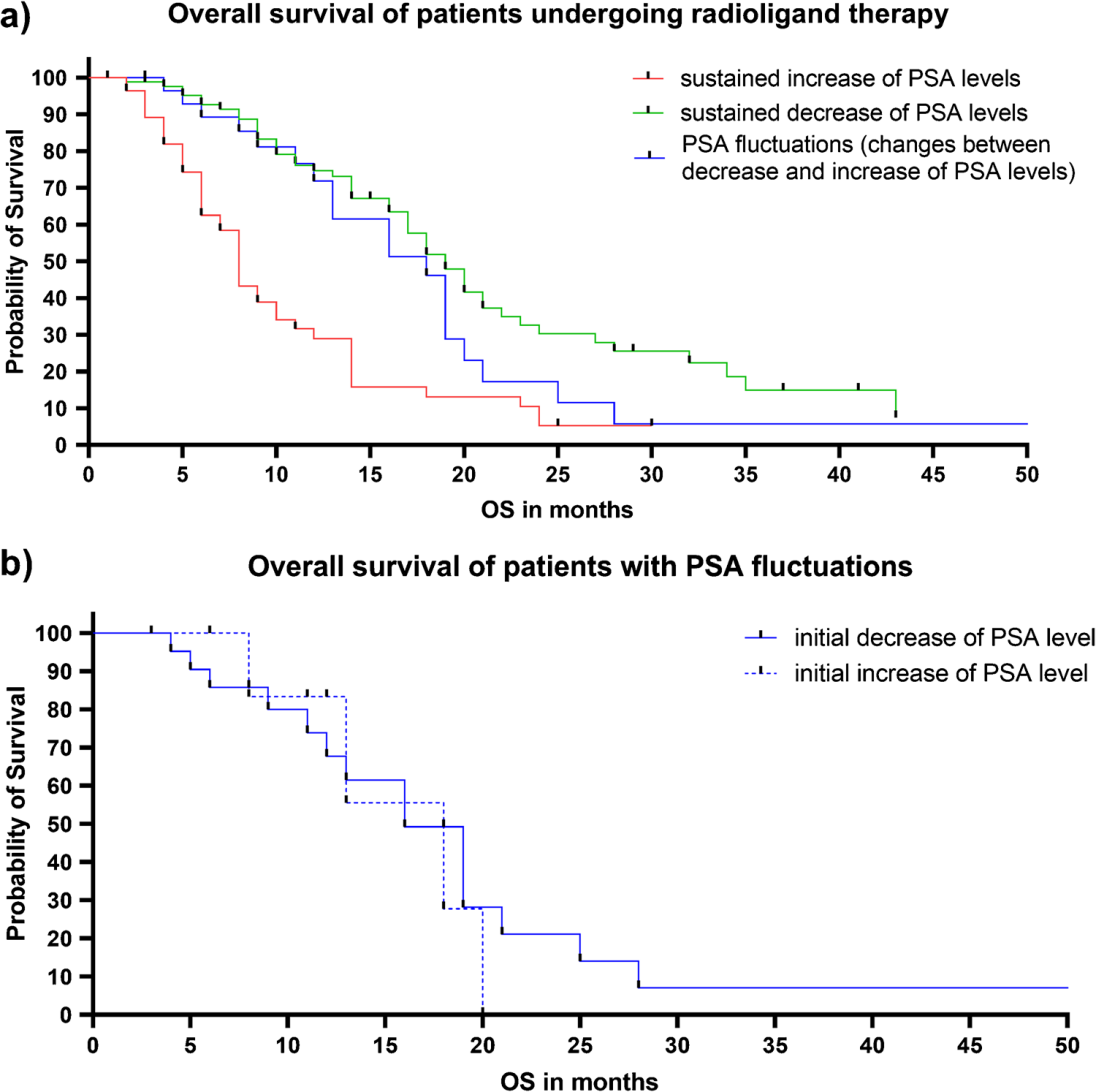
**a** Median overall survival (OS) of patients with sustained decrease in PSA levels (green) was significantly longer when compared to men with sustained increase of PSA levels (red) (19 vs. 8 months; HR 0.35, 95% CI 0.22–0.56; P < 0.001). Median OS of patients with PSA fluctuations (i.e., change between increase or decrease of PSA levels; blue) was significantly longer compared to patients with sustained increase of PSA levels (red) (18 vs. 8 months; HR 0.49, 95% CI 0.30–0.80; P < 0.01). However, there was no significant difference in OS between patients with PSA fluctuations (blue) to patients with sustained decrease in PSA levels (green) (18 vs. 19 months; HR 1.4, 95% CI 0.78–2.49; P = 0.20), thereby indicating that patients with tumor marker oscillations should continue treatment. **b** In the subgroup of patients with PSA fluctuations (i.e., changes between increase or decrease), the median OS did not differ significantly between patients with initial decrease or initial increase of PSA levels (16 vs. 18 months; HR 1.2, 95% CI 0.38–4.05; P = 0.68)

Median OS of patients with sustained decrease in PSA levels was significantly longer when compared to patients with sustained increase of PSA levels (19 vs. 8 months; HR 0.35, 95% CI 0.22–0.56; *P* < 0.001). Patients with PSA fluctuations showed a significantly longer median OS compared to patients with sustained increase of PSA levels (18 vs. 8 months; HR 0.49, 95% CI 0.30–0.80; *P* < 0.01), but were comparable to those patients with sustained decrease in PSA levels (18 vs. 19 months; HR 1.4, 95% CI 0.78–2.49; *P* = 0.20; Fig. 4a).

In the subgroup of patients experiencing PSA fluctuations during follow-up, median OS did not differ significantly between patients with initial decrease (followed by an increase) or late responders (16 vs. 18 months; HR 1.2, 95% CI 0.38–4.05; *P* = 0.68; Fig. 4b).

Similar findings were also observed for a subgroup analysis investigating patients treated with either [^177^Lu]Lu-PSMA I&T or [^177^Lu]Lu-PSMA-617 (Supplementary Fig. 1).

## Discussion

In the present analysis including 176 patients under PSMA-directed RLT, we demonstrated that patients with signs of response or disease stabilization have longer OS compared to patients with PD, when current PCWG3 criteria are applied [16]. Independent of those criteria, we demonstrated that patients with an initial decrease in PSA levels are highly likely to sustain this decrease over four cycles of therapy and that this trend was linked to prolonged OS. Men with initial PSA increase, however, exhibited a continued rise of their tumor marker levels during follow-up, ultimately experiencing shorter survival. PSA fluctuations, defined as change after initial decrease or increase, occurred relatively frequently and survival rates were comparable to the beneficial outcome seen in the group of patients with sustained PSA decline. Thus, such tumor marker oscillations should not lead to discontinuation of PSMA-RLT, in particular in patients with initial decline followed by a rise in PSA levels, as this scenario may be commonly mistaken as disease progression, especially in the absence of radiological progression.

Recommendations of response assessment for anti-tumor treatments in men with PC have been established by PCWG3 [16]. Using those commonly applied response criteria, we demonstrated that patients with PD (category 6) had shorter survival when compared to men with signs of response or disease stabilization (categories 1–5; Fig. 1). As the latter categories also incorporate patients with PSA fluctuations, PCWG3-based assessment revealed then identical findings similar to our analyses using a simplified definition of PSA response (Fig. 4a). Of note, there were also no significant differences between patients within the categories 1–5 for OS, and thus, individuals with PSA fluctuations had also comparable survival benefits to those subjects with PCWG3-defined sustained response (category 1). Taken together, independent of defining PSA response, patients with tumor marker oscillations exhibit comparable survival to those individuals which always show biochemical response under RLT.

Our analysis indicates that patients exhibiting an initial PSA response sustain this trend over multiple cycles, while in patients with initial increase of PSA, there is a high likelihood of a continued rise of tumor marker levels. These findings corroborate previous results, in particular for [^177^Lu]Lu-PSMA-617 [4, 10]. In a matched-pair analysis, we have also demonstrated no relevant survival differences in patients treated with either [^177^Lu]Lu-PSMA-617 or -I&T [13]. Thus, given the results of this previous and the present study providing comparable percentages of patients maintaining an initial PSA decrease or increase, the treating physician can be confident that these trends are to be expected for both PSMA-targeted agents [13]. These considerations are further fueled by a sub-analysis (Supplementary Fig. 1), where we also observed comparable results for patients treated with [177Lu]Lu-PSMA-617 or -I&T, as seen for the entire cohort.

Among the entire cohort, we observed a late response (i.e., PSA increase followed by a decrease) in only 7/176 (4%). As such, enrolling a substantially larger cohort, we corroborated previous findings by Gafita et al., which also observed this phenomenon in approximately 1% of their patients [5]. Previous work, however, reported on approximately 8% [4], which were comparable to the ones reported by Soydal et al. with 10% and up to 17% by Rahbar and coworkers (% derived by extrapolation taking the entire cohorts into account) [10, 11]. As a possible explanation, the number of included individuals was lower relative to the present work and unifying definitions along with prospective studies further evaluating this aspect in mCRPC patients under RLT are warranted.

Within the subgroup showing PSA fluctuations, the majority of men experienced an increase of PSA levels after initial decrease (> 75%). In clinical routine, this finding is normally explained by disease progression. However, these patients still showed a longer OS, which was comparable to those with initial and sustained decrease of PSA levels and significantly longer when compared to patients with continuous increase (Fig. 4a). These results suggest that patients initially showing a decrease in their PSA levels may rather not discontinue treatment, in particular if they maintain this trend or even when they exhibit a PSA rise during follow-up, especially in the absence of radiological progression. Moreover, results of the present study are also in line with other reports on prostate-specific therapies and PSA flare under therapy, including chemotherapy, anti-hormonal treatment, or the use of alpha emitters primarily targeting osseous disease manifestations. For those therapies, early discontinuation due to transient PSA rises was also not recommended [7–9]. In addition, an initial (or later) PSA response might also emerge as a powerful prognosticator for OS, further supporting previous analyses on a positive predictive value of an initial PSA response after the first treatment cycle [4–6]. Nonetheless, PSA monitoring can only partially address the complexity of responding to PSMA-directed RLT, and thus, other markers of response may be also of relevance, e.g., circulating tumor cells [17]. Those established or currently emerging biomarkers may be less prone to oscillations as observed for PSA in the present investigation.

Our results are limited due to the retrospective design. Nonetheless, the PSA values were consistently assessed on a regular basis and time-points of blood samples were in accordance to current procedure guidelines [18]. Relative to previous reports, the number of patients is high and consisted of two cohorts treated with the two most commonly used radiopharmaceuticals, which have been reported to be comparable in their therapeutic efficacy [13]. In addition, we did also not observe any differences in the patient’s characteristics between both cohorts in our study (Table 1). In our cohort, only a few patients had documented post-RLT treatments (four patients had radiotherapy, eight had chemotherapy, two had abiraterone, and one subject had received olaparib). However, given the fact that PSMA-RLT is currently considered the last-line treatment option [18], we assume that the possible impact of follow-up therapies on survival may have been rather limited. In addition, future studies may also perform sub-analyses for patients which had received the identical treatment algorithm prior to RLT, preferably in a larger number of subjects. Moreover, radiological assessment is also used for identifying patients that do not respond to RLT and combined approaches by PSA measurements and PSMA-PET/CT have already shown to be prognostic for OS [19]. Thus, the herein observed PSA fluctuations should also be evaluated together with such PET/CT-based response evaluations. Further investigations may then also focus on patients which showed PSA increase after initial decrease and whether those individuals also experience radiological progression.

## Conclusions

Initial increase or decrease of PSA levels is sustained in the majority of patients undergoing RLT. Sustained PSA decrease was linked to prolonged survival and men with PSA fluctuations under treatment experienced comparable survival benefits. Thus, such frequent tumor marker oscillations should not lead to an early discontinuation of PSMA-RLT. This may apply in particular to scenarios that could be mistaken as progressive disease, such as in patients experiencing initial decline early in the treatment course followed by rise of PSA levels during follow-up, especially in the absence of radiological progression.

## Supplementary Information

Below is the link to the electronic supplementary material.

ESM 1 Supplementary Figure 1: **(a):** In patients treated with [^177^Lu]Lu-PSMA I&T, median overall survival (OS) of patients with sustained decrease in PSA levels (green) was significantly longer when compared to men with sustained increase of PSA levels (red) (20 vs. 9 months; HR 0.34, 95% CI 0.18-0.65; P<0.001). Median OS of patients with PSA fluctuations (i.e., change between increase or decrease of PSA levels; blue) was significantly longer compared to patients with sustained increase of PSA levels (red) (19 vs. 9 months; HR 0.34, 95% CI 0.16-0.70; P=0.01). However, there was no significant difference in OS between patients with PSA fluctuations (blue) to patients with sustained decrease in PSA levels (green) (19 vs. 20 months; HR 1.064, 95% CI 0.41-2.74; P=0.90). **(b):** Similar findings were observed in the sub-group treated with [^177^Lu]Lu-PSMA-617. Median OS of patients with sustained decrease in PSA levels (green) was significantly longer when compared to men with sustained increase of PSA levels (red) (18 vs. 5 months; HR 0.34, 95% CI 0.16-0.70; P<0.001). Median OS of patients with PSA fluctuations (i.e., change between increase or decrease of PSA levels; blue) was significantly longer compared to patients with sustained increase of PSA levels (red) (16 vs. 5 months; HR 0.52, 95% CI 0.26-1.05; P<0.05). Again, there was no significant difference in OS between patients with PSA fluctuations (blue) to patients with sustained decrease in PSA levels (green) (16 vs. 18 months; HR 1.61, 95% CI 0.79-3.32; P<0.13). (PNG 174 kb)

High resolution image (TIF 886 kb)

## Data Availability

Detailed information on the results presented in this study is available on reasonable request from the corresponding author.
