# Supplementary figures and images for: mCRPC patients with PSA fluctuations under radioligand therapy have comparable survival benefits relative to patients with sustained PSA decrease
Source: Eur J Nucl Med Mol Imaging. 2022 Jul 19;49(13):4727–35. doi: 10.1007/s00259-022-05910-w (PMC9606086; doi:10.1007/s00259-022-05910-w)

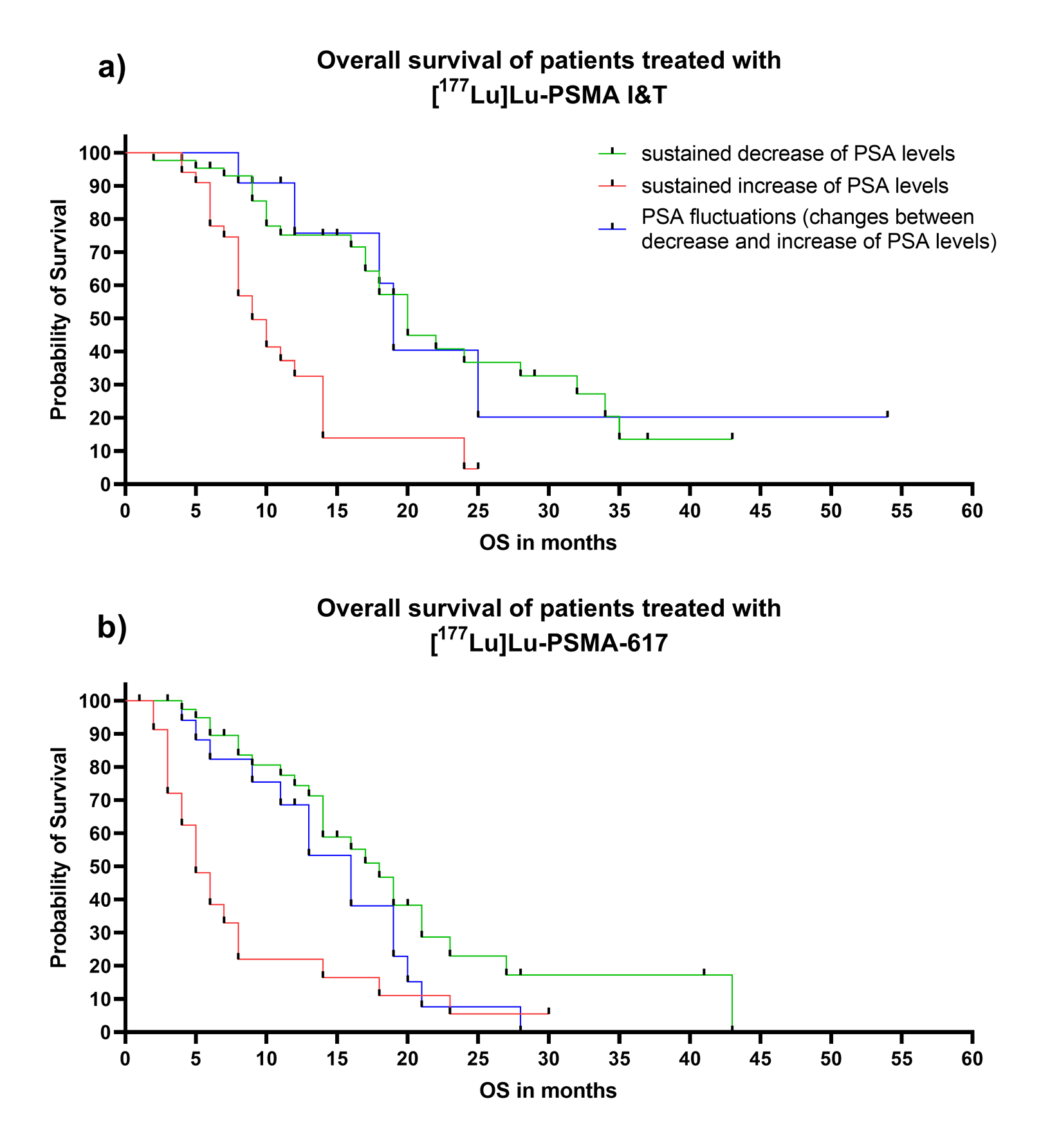

Supplement: Supplementary file 1 — Supplementary Figure 1: (a): In patients treated with [177Lu]Lu-PSMA I&T, median overall survival (OS) of patients with sustained decrease in PSA levels (green) was significantly longer when compared to men with sustained increase of PSA levels (red) (20 vs. 9 months; HR 0.34, 95% CI 0.18-0.65; P<0.001). Median OS of patients with PSA fluctuations (i.e., change between increase or decrease of PSA levels; blue) was significantly longer compared to patients with sustained increase of PSA levels (red) (19 vs. 9 months; HR 0.34, 95% CI 0.16-0.70; P=0.01). However, there was no significant difference in OS between patients with PSA fluctuations (blue) to patients with sustained decrease in PSA levels (green) (19 vs. 20 months; HR 1.064, 95% CI 0.41-2.74; P=0.90). (b): Similar findings were observed in the sub-group treated with [177Lu]Lu-PSMA-617. Median OS of patients with sustained decrease in PSA levels (green) was significantly longer when compared to men with sustained increase of PSA levels (red) (18 vs. 5 months; HR 0.34, 95% CI 0.16-0.70; P<0.001). Median OS of patients with PSA fluctuations (i.e., change between increase or decrease of PSA levels; blue) was significantly longer compared to patients with sustained increase of PSA levels (red) (16 vs. 5 months; HR 0.52, 95% CI 0.26-1.05; P<0.05). Again, there was no significant difference in OS between patients with PSA fluctuations (blue) to patients with sustained decrease in PSA levels (green) (16 vs. 18 months; HR 1.61, 95% CI 0.79-3.32; P<0.13). (PNG 174 kb) [file 259_2022_5910_Fig5_ESM.png]

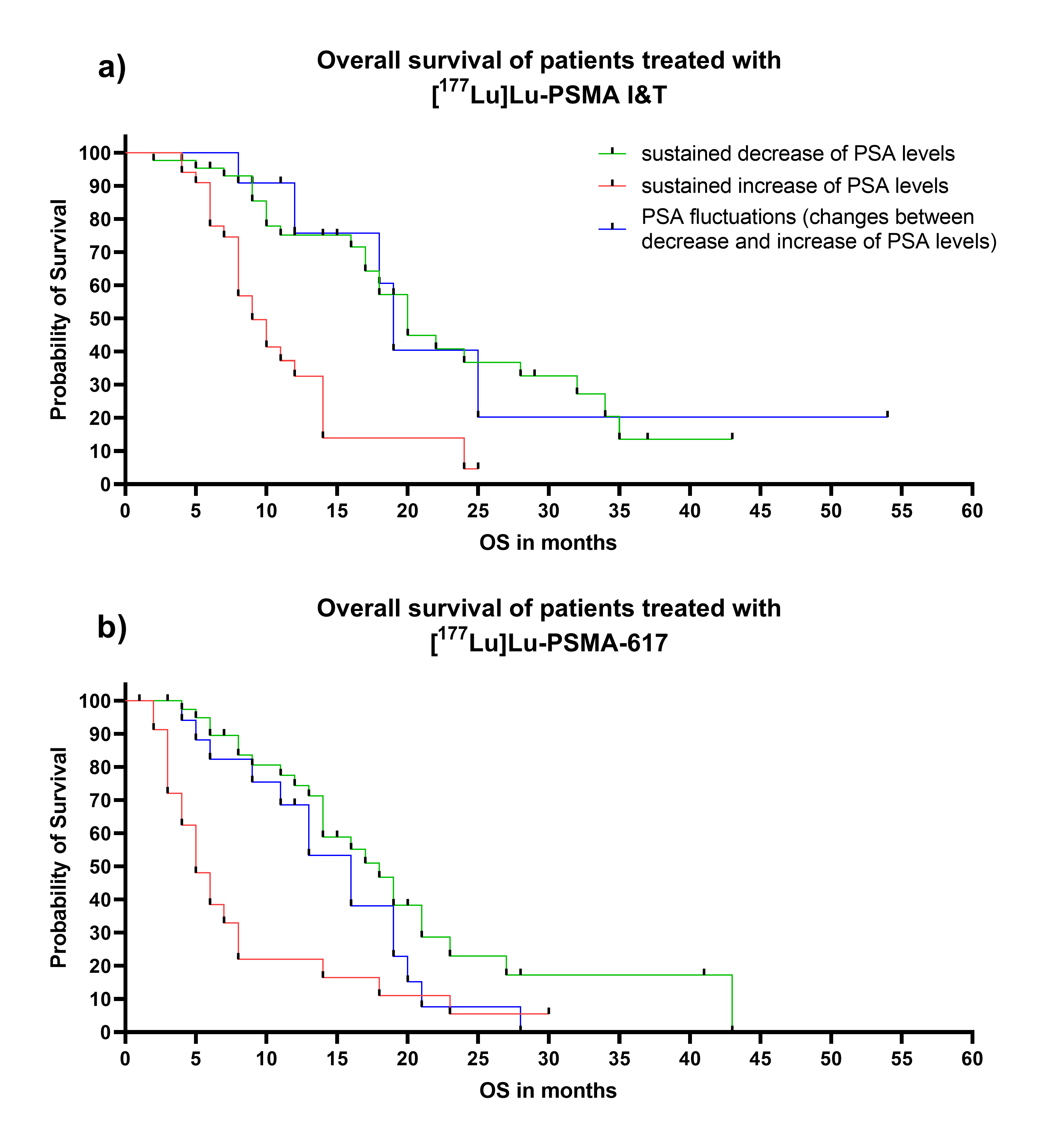

Supplement: Supplementary file 2 — High resolution image (TIF 886 kb) [file 259_2022_5910_MOESM1_ESM.tif]
